# Extreme temperature exposure and urolithiasis: A time series analysis in Ganzhou, China

**DOI:** 10.3389/fpubh.2022.1075428

**Published:** 2022-12-14

**Authors:** Zhijin Li, Yanlu Li, Xiaoning Wang, Guoliang Liu, Yanbin Hao

**Affiliations:** ^1^Department of Health Statistics, School of Public Health and Health Management, Gannan Medical University, Ganzhou, China; ^2^Department of Urology, First Affiliated Hospital of Gannan Medical University, Ganzhou, China; ^3^Jiangxi Engineering Technology Research Center of Calculi Prevention, Gannan Medical University, Ganzhou, China; ^4^School of Medical Information Engineering, Gannan Medical University, Ganzhou, China

**Keywords:** extreme temperature, urolithiasis, warm effect, cold effect, time series analysis

## Abstract

**Background:**

Ambient temperature change is a risk factor for urolithiasis that cannot be ignored. The association between temperature and urolithiasis varies from region to region. Our study aimed to analyze the impact of extremely high and low temperatures on the number of inpatients for urolithiasis and their lag effect in Ganzhou City, China.

**Methods:**

We collected the daily number of inpatients with urolithiasis in Ganzhou from 2018 to 2019 and the meteorological data for the same period. The exposure-response relationship between the daily mean temperature and the number of inpatients with urolithiasis was studied by the distributed lag non-linear model (DLNM). The effect of extreme temperatures was also analyzed. A stratification analysis was performed for different gender and age groups.

**Results:**

There were 38,184 hospitalizations for urolithiasis from 2018 to 2019 in Ganzhou. The exposure-response curve between the daily mean temperature and the number of inpatients with urolithiasis in Ganzhou was non-linear and had an observed lag effect. The warm effects (30.4°C) were presented at lag 2 and lag 5–lag 9 days, and the cold effects (2.9°C) were presented at lag 8 and lag 3–lag 4 days. The maximum cumulative warm effects were at lag 0–10 days (cumulative relative risk, CRR = 2.379, 95% CI: 1.771, 3.196), and the maximum cumulative cold effects were at lag 0–5 (CRR = 1.182, 95% CI: 1.054, 1.326). Men and people between the ages of 21 and 40 were more susceptible to the extreme temperatures that cause urolithiasis.

**Conclusion:**

Extreme temperature was correlated with a high risk of urolithiasis hospitalizations, and the warm effects had a longer duration than the cold effects. Preventing urolithiasis and protecting vulnerable people is critical in extreme temperature environments.

## Introduction

Global climate change is becoming more and more intense. The health effects of meteorological factors have become a research hotspot. The impact of temperature on people's health has attracted increasing attention. Studies in China and other countries (1–3) showed that temperature change affects the occurrence of many diseases, including urolithiasis. Urolithiasis is a common disease in urology, which includes diseases such as kidney stones and ureteral stones. The prevalence of urolithiasis in the Asian population was about 1–19.1%, with a high recurrence rate of 21–53% within 3–5 years, and the lifetime recurrence risk was about 60–80% (4). Due to global warming, the incidence of urolithiasis will become more severe. One study estimated that the number of kidney stone cases related to climate change in the USA would increase by 1.6 to 2.2 million by 2050 (5).

Some studies confirmed the correlation between ambient temperature change and the onset of urolithiasis. Geraghty et al. (6) pointed out that the incidence of urolithiasis usually showed obvious seasonality, and the incidence of urolithiasis increases with the increase of monthly mean temperature. Moreover, the exposure-response effects associated with temperature and urolithiasis were different under high-temperature conditions. Studies have shown that men are more affected by rising ambient temperatures (7). A few studies showed that extremely low temperatures increase the risk of urolithiasis (8, 9). Furthermore, the influence of temperature on urolithiasis has obvious regional specificity (10), which may be related to the dietary habits, lifestyle, demographic characteristics, and other environmental factors of the local population (11–13).

The incidence of urolithiasis in southern China was significantly higher than that in northern China (10, 12), and Ganzhou City in Jiangxi Province was a high-risk area for urolithiasis in southern China. Studying the impact of temperature on urolithiasis in Ganzhou helped mitigate the risk of disease caused by climate change in high-risk areas. It was significant to develop effective prevention and control measures and targeted strategies. In this study, we used the distributed lag non-linear model (DLNM) to analyze the impact of extremely high and low temperatures on the number of inpatients for urolithiasis and its lag effect. Further, the inpatients were stratified by gender and age to provide a scientific basis for understanding vulnerable people affected by extreme temperatures.

## Materials and methods

### Study location

Ganzhou City (24°29′-27°09′ N; 113°54′-116°38′ E) is located in the south of Jiangxi Province and the southeast of China. Ganzhou is a prefecture-level city with the largest area (39,379.64 square kilometers) and largest population (8.98 million) in Jiangxi Province. It is located in the subtropical monsoon climate zone, with the climatic characteristics of prevailing winter and summer monsoons and concentrated rainfall in spring and summer.

### Data collection

The data on urolithiasis hospitalizations from 2018 to 2019 were obtained from the Diagnosis Related Groups (DRGs) data analysis platform of the Jiangxi Provincial Health Commission. These cases were from 69 hospitals (16 tertiary and 53 secondary hospitals) in Ganzhou. The spatial distribution of hospitals from which hospitalization records came is shown in Supplementary Figure 1. Information on each inpatient included the patient's gender, age, main discharge diagnosis name, main discharge diagnosis code, and other confidential personal information. Urolithiasis cases were selected according to the ICD-10 codes (N20–N23) and the main discharge diagnosis name by text search. Almost all urolithiasis patients were hospitalized due to low back pain, hematuria, infection, and other acute symptoms leading to admission for treatment.

The meteorological data for the same period were from the National Meteorological Science Data Center (http://data.cma.cn/). There were four national basic meteorological monitoring stations (Ganxian, Xunwu, Ningdu, and Longnan) in Ganzhou City. Meteorological variables included the daily mean temperature (°C), maximum temperature (°C), minimum temperature (°C), average relative humidity (%), and cumulative rainfall (mm). We calculated the mean values for each meteorological variable by using the complete data from four stations.

### Statistical analysis

A distributed lag non-linear model (DLNM) was used to estimate the exposure-response relationship between the daily mean temperature and the number of inpatients with urolithiasis. DLNM was originally developed to study the association between temperature and mortality. DLNM could simultaneously model the non-linear and lag effects between exposure and adverse outcomes (14, 15). The effect of extreme temperatures on urolithiasis hospitalizations was mainly evaluated using two indicators: relative risk (RR) and cumulative relative risk (CRR) (16, 17). Based on previous studies, the lag period was 10 days, and the reference temperature was 10°C (8, 18). First, the RR of urolithiasis hospitalizations at each lag day after extreme temperature exposure (lag-response relationship) during the 10 days was estimated. Each day lag was expressed at lag*t* (*t* = 0~10). Second, we summarized the RRs for lag days (lag*0* – *t*) to estimate the CRR of urolithiasis hospitalizations after temperature exposure (cumulative exposure-response relationship) (8). Considering the overdispersion of the daily number of urolithiasis hospitalizations, we used “quasi-Poisson” as the link function of the model. The potential confounding factors, including relative humidity, rainfall, time trends, and day of the week, were controlled in the constructed model. The model was expressed as follows:


$$\begin{array}{l} \log\left[E\left(Y_{t}\right)\right]=\alpha +cb\left(Temp_{t,l}\right)+NS\left(time,df\right)+\\ \;NS\left(h,df\right)+NS\left(r,df\right)+DOW_{t} \end{array}$$

Here, *Y*_*t*_ is the daily number of inpatients for urolithiasis, α is the intercept, and *cb*(*Temp*_*t, l*_) is the cross-basis matrix of the daily mean temperature and lag time. Expose-response fitting was performed using a natural cubic spline function with node locations in the 50th and 90th percentiles. A natural cubic spline function was also used for expose-lag fitting, and logarithmic equal spacing was used for node location setting. *NS* was a natural cubic spline function, *df* was the degree of freedom, *time* was a time variable, *DOW* was a dummy variable that controlled the day of the week effect.

According to previous studies (8, 18) and the minimum principle of the quasi-Akaike information criterion (q-AIC), the lag duration was determined to be 10 days, and *r* and *h* were included in the model with a natural cubic spline function (*df* = 3), and the *df* of time was selected to be 7/year. Taking the daily mean temperature of 10°C as the reference temperature, the predicted value range was 0~32°C with an interval of 0.1°C. Warm and cold effects on urolithiasis hospitalizations were estimated at the ambient temperature values of P99 (30.4°C) and P1 (2.9°C) (19). To identify vulnerable people, we further conducted subgroup analyses by gender (male and female) and age groups (0–20, 21–40, 41–60, and >60 years old).

Excel 2019 and R4.0.2 software were used for data collection and analysis. The “dlnm” package was used for DLNM modeling, the “cross basis” function was used to build the cross basis, the “glm” function was used to fit the model, the “crossred” function was used to estimate the relative risk (RR) and its cumulative value (CRR), and the “plot” function was used to visualize the results. Statistical significance was considered by *p* < 0.05.

### Sensitivity analysis

We performed sensitivity analyses to confirm the robustness of the results by changing the degree of freedom of the time trend (6–9/year) in the model. In addition, we used P95 and P5 of ambient temperature values to redefine extremely high and low temperatures and compare the results of different definitions.

## Results

Table 1 shows the descriptive statistics of daily hospital admissions for urolithiasis and meteorological variables in Ganzhou from 2018 to 2019. A total of 38,184 urolithiasis hospitalizations were reported, and the daily average number of inpatients was 52. According to the resident population (CEIdata, https://ceidata.cei.cn/jsps/Default), the 2-year average annual hospitalization rate was 214.8 per 100,000. There were 21,602 (56.6%) men and 16,316 (42.7%) women; the men-to-women sex ratio was 1.32:1, and the other 266 (0.7%) cases had not specified their gender. The age group between 41 and 60 years old had the largest number of cases (19,448 cases), accounting for 50.93%. During the study period, the average values of the daily mean temperature, maximum temperature, minimum temperature, rainfall, and relative humidity were 19.9, 25.3, 16.3°C, 4.4 mm, and 76.5%, respectively.

**Table 1:** Summary of daily urolithiasis hospitalizations and meteorological variables in Ganzhou from 2018 to 2019.

**Variables**	**Min**	**P5**	**P25**	**Median**	**P75**	**P95**	**Max**	** $\bar{x}\pm SD$ **
Total urolithiasis cases	2	26	39	50	63	88	134	52 ± 19
**Gender**
Male	0	14	22	28	36	52	76	30 ± 12
Female	1	9	16	21	28	38	62	22 ± 9
**Age**
0–20 years old	0	0	1	2	4	6	10	3 ± 2
21–40 years old	1	4	8	10	13	20	32	11 ± 5
41–60 years old	0	11	19	25	32	46	76	27 ± 11
>60 years old	0	5	8	11	15	23	34	12 ± 6
**Meteorological variables**
Mean temperature (°C)	1.2	6.6	14.0	20.8	26.9	29.7	31.4	19.9 ± 7.6
Maximum temperature (°C)	2.3	9.6	19.5	26.7	32.5	35.8	37.6	25.3 ± 8.4
Minimum temperature (°C)	−3.0	3.1	10.2	17.0	23.6	25.4	26.9	16.3 ± 7.5
Rainfall (mm)	0.0	0.0	0.0	0.3	3.9	23.8	76.8	4.4 ± 9.2
Relative humidity (%)	34.5	55.8	69.6	77.3	85.0	92.9	97.0	76.5 ± 11.1

Min, minimum; Max, maximum; $\bar{x},\;$mean; SD, standard deviation. P25 denoted the 25th percentile, P75 denoted the 75th percentile, P90 denoted the 90th percentile, and P95 denoted the 95th percentile.

**Figure 1:**
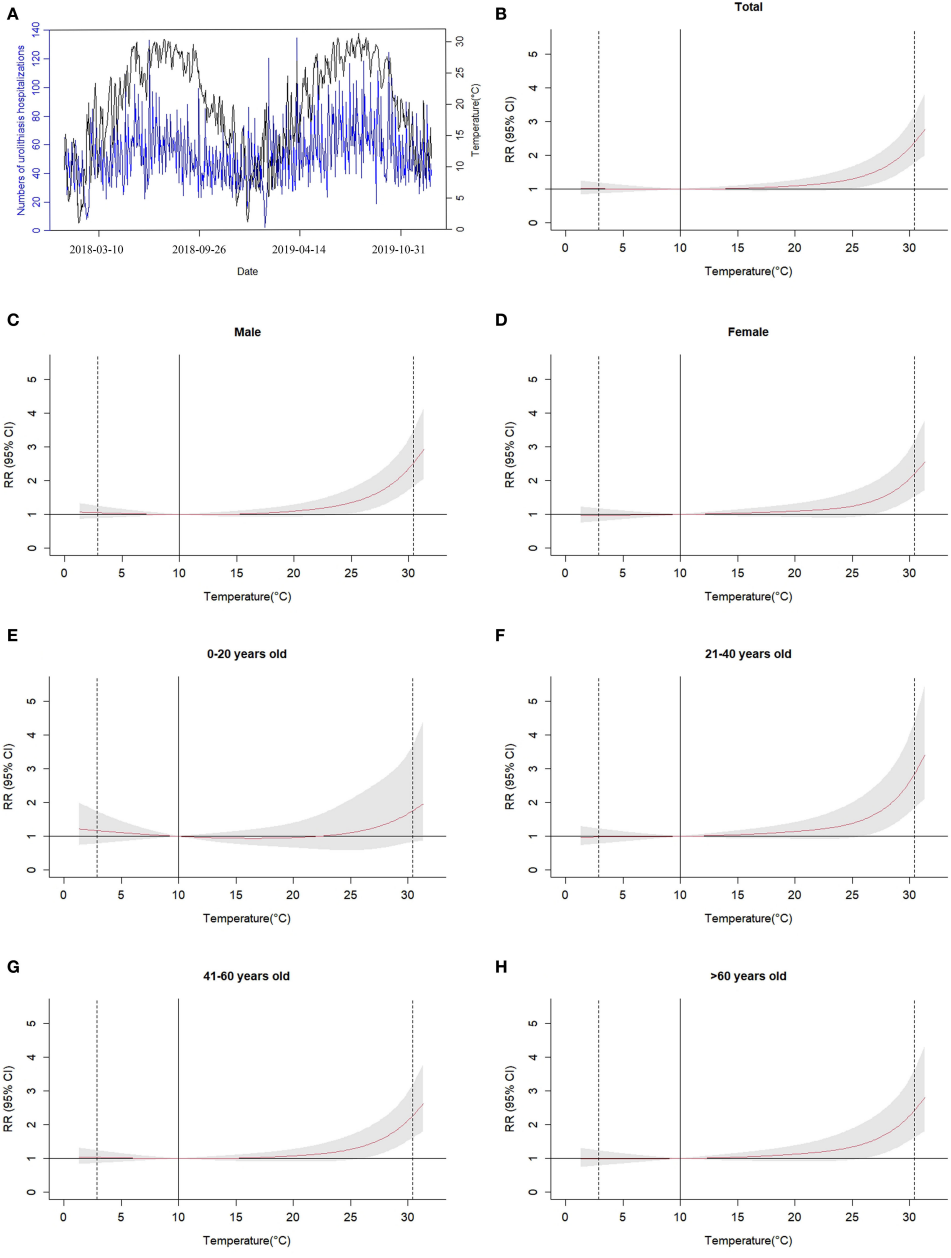
Temporal trend **(A)** and effect curves **(B–H)** of the daily mean temperature on the number of inpatients with urolithiasis in Ganzhou City during the 10 days lag period, relative to 10°C. The vertical solid line at 10°C represented the reference temperature. The two vertical dotted lines represent P1 (2.9°C) and P99 (30.4°C) of the daily mean temperature, respectively. The red curve was RR, and the surrounding gray area was 95% CI.

We could see the temporal dependent trend changes and exposure-response curve between the daily mean temperature and risk of urolithiasis hospitalizations (Figure 1). The number of hospitalizations for urolithiasis increased rapidly during the high-temperature period. The effect curve of the daily mean temperature on the number of inpatients with urolithiasis was nearly “*J*” type.

Taking P99 (30.4°C) of the daily mean temperature in Ganzhou from 2018 to 2019 as the extremely high temperature and P1 (2.9°C) as the extremely low temperature, we analyzed the warm and cold effects on urolithiasis hospitalizations. Table 2 shows the relative risk (RR) of urolithiasis hospitalizations related to extreme temperatures. Table 3 shows the cumulative relative risk (CRR) of urolithiasis hospitalizations associated with extreme temperatures. Compared with 10°C, the warm effects of ambient temperature on the hospitalizations of urolithiasis changed to the maximum at lag 2 (RR = 1.091, 95% CI: 1.002, 1.187) and lasted significantly for 5 days (lag 5–lag 9). The cold effect of the ambient temperature on the hospitalizations for urolithiasis changed more at lag 3 and lag 4, lasting for 2 days. The maximum effect was at lag 3 (RR = 1.089, 95% CI: 1.016, 1.167). The cumulative warm effect occurred at lag 0–10 with the largest effect (CRR = 2.379, 95% CI: 1.771, 3.196). The cumulative cold effect occurred from lag 0–3 to lag 0–6 and reached the maximum at lag 0–5 (CRR = 1.182, 95% CI: 1.054, 1.326).

**Table 2:** Warm and cold effects of temperature on urolithiasis hospitalizations at different lag days.

**Lag day**	**Urolithiasis hospitalizations**	
	**Warm effect**	**Cold effect**
lag0	1.089 (0.845, 1.403)	1.050 (0.907, 1.215)
lag1	1.241 (0.993, 1.551)	0.939 (0.815, 1.082)
lag2	1.091 (1.002, 1.187)*	1.047 (0.990, 1.108)
lag3	1.023 (0.922, 1.135)	1.089 (1.016, 1.167)*
lag4	1.038 (0.973, 1.107)	1.049 (1.006, 1.094)*
lag5	1.067 (1.016, 1.121)*	1.002 (0.969, 1.037)
lag6	1.082 (1.019, 1.149)*	0.975 (0.936, 1.017)
lag7	1.086 (1.028, 1.146)*	0.964 (0.929, 1.000)
lag8	1.079 (1.036, 1.124)*	0.963 (0.939, 0.987)*
lag9	1.067 (1.011, 1.127)*	0.970 (0.937, 1.004)
lag10	1.052 (0.954, 1.160)	0.980 (0.918, 1.047)

Warm effect: 30.4°C, cold effect: 2.9°C, *p < 0.05.

**Table 3:** Cumulative warm and cold effects of temperature on hospitalization of urolithiasis.

**Cumulative lag days**	**Urolithiasis hospitalizations**	
	**Warm effect**	**Cold effect**
lag0–0	1.089 (0.845, 1.403)	1.050 (0.907, 1.215)
lag0–1	1.351 (1.110, 1.645)*	0.986 (0.882, 1.102)
lag0–2	1.473 (1.215, 1.787)*	1.033 (0.926, 1.152)
lag0–3	1.507 (1.242, 1.829)*	1.124 (1.010, 1.251)*
lag0–4	1.564 (1.263, 1.938)*	1.179 (1.049, 1.325)*
lag0–5	1.670 (1.339, 2.082)*	1.182 (1.054, 1.326)*
lag0–6	1.807 (1.438, 2.272)*	1.153 (1.027, 1.294)*
lag0–7	1.962 (1.536, 2.506)*	1.111 (0.980, 1.260)
lag0–8	2.118 (1.634, 2.745)*	1.070 (0.935, 1.225)
lag0–9	2.261 (1.726, 2.961)*	1.038 (0.902, 1.194)
lag0–10	2.379 (1.771, 3.196)*	1.017 (0.868, 1.191)

Warm effect: 30.4°C, cold effect: 2.9°C, *p < 0.05.

For different gender and age groups, we also obtained the cumulative effect of temperature with different lag days on the risk of urolithiasis hospitalizations. Table 4 shows the cumulative effects of lag for 10 days at high temperature (30.4°C) and those of lag for 5 days at low temperature (2.9°C). Both men and women had significant cumulative warm and cold effects on urolithiasis hospitalizations. The warm effect values were higher than the cold effect values, and the effects on men were higher. For different age groups, the temperature had significant warm effects on people over 21 years old. However, those between the ages of 21 and 60 were particularly vulnerable to cold. The population of 21–40 years old had the highest cumulative warm effect values (CRR = 2.835, 95% CI: 1.831, 4.390) and cumulative cold effect values (CRR = 1.216, 95% CI: 1.034, 1.429).

**Table 4:** Cumulative warm and cold effects of temperature on urolithiasis inpatients of different genders and ages.

**Group**	**Warm effect (lag0–10)**	**Cold effect (lag0–5)**
**Gender**
Male	2.516 (1.824, 3.472)*	1.199 (1.059, 1.359)*
Female	2.195 (1.528, 3.153)*	1.156 (1.004, 1.331)*
**Age**
< 20 years old	1.751 (0.831, 3.688)	1.136 (0.843, 1.531)
21–40 years old	2.835 (1.831, 4.390)*	1.216 (1.034, 1.429)*
41–60 years old	2.263 (1.610, 3.181)*	1.210 (1.061, 1.381)*
>60 years old	2.415 (1.617, 3.607)*	1.091 (0.926, 1.286)

Warm effect: 30.4°C, cold effect: 2.9°C, *p < 0.05.

The sensitivity analysis showed that the results were still robust after changing the *df* of the time trend (Supplementary Figure 2). In addition, applying P95 and P5 of daily mean temperatures to define extremely high and low temperatures showed fairly close results (Supplementary Figures 3, 4).

## Discussion

In this study, we used the DLNM model to analyze the impact of extreme temperatures on urolithiasis hospitalizations in Ganzhou from 2018 to 2019. The association between the daily mean temperature and the number of urolithiasis inpatients was nonlinear, had a “*J*” shape, and had a certain lag effect. Both the warm effects at high temperatures and the cold effect at low temperatures existed and increased the risk of hospitalization for urolithiasis. Different lag times and durations of warm and cold effects on urolithiasis hospitalizations were found; high temperatures had larger and more sustained effects than low temperatures.

We found that, with the increase in temperature, the effect value of hospitalization risk of urolithiasis was increasing, which was consistent with studies on the association between ambient temperature and urolithiasis (8, 20–22). In addition to confirming the warm effects of high temperatures on urolithiasis hospitalization, we also found the effects of low temperatures. Fewer studies found a risk of urolithiasis from cold temperatures. A study analyzed the characteristics of emergency urolithiasis patients in South Carolina and found that 63.2% of patients lived in cold areas, while only 36.8% lived in warm ones (18). A time series analysis in the USA indicated that Atlanta (<2°C), Chicago, and Philadelphia (<10°C) had an increased risk of kidney stones (8).

The biological mechanism of high- or low-temperature-induced urolithiasis was still unclear. When in a high-temperature environment, the human body warms, which induces sweating, decreases extracellular fluid, increases vasopressin secretion, urine concentration, calcium, and uric acid concentrations, and accelerates the formation of stones (2, 23). Further, another research showed that a large amount of sunlight in a warm climate leads to higher vitamin D levels, increasing urinary calcium excretion (7). People might drink less when the temperature is low because they perspire less and feel less thirsty. Additionally, people would prefer to stay warm indoors and do less exercise. The reduction of exercise and water intake were risk factors for the development of urolithiasis (24). However, the effect and risk of temperature change on the occurrence of urolithiasis in the population still needed to be verified through epidemiological observational study research, which helped better understand how temperature change could induce urinary diseases, publicize, and educate the public about the risk factors related to urolithiasis in combination with climate change (25), and finally, more actively reduce the negative effects of extreme temperature exposure (3).

The study found a higher risk of hospitalization for urolithiasis within 10 days after extreme heat. Fletcher et al. (26) found that the effect of high temperature was mainly within a lag of 3 days. Chi et al. (20) found that there was a positive correlation between the daily mean temperatures and the daily incidence of urolithiasis, and the lag effect was within 5 days. In Seoul, the effects of high temperatures on urolithiasis were also found to be mainly within a lag of 5 days (27). A case study in Vietnam observed elevated point estimates for lag 0 to lag 6 days (28). Tasian et al. (8) found that the impact of high temperatures on urolithiasis in some metropolitan areas of the United States remained at risk within a lag of 20 days. The lag period of urolithiasis was different in different regions, and further research was needed to explore the causes of this difference. The lagging effect of extreme temperatures on urolithiasis hospitalizations suggested that, to achieve better public health prevention, residents should not only pay attention to the need for protection on the day of extreme temperature exposure but also protect against the long-term repercussions of such exposure. We suggested future studies should consider the lag period between extreme temperatures and disease, which was selected due to regional and disease differences.

Stratified by gender, the cumulative warm and cold effects on men were higher than those on women. Fakheri et al. (23) and Brog et al. (2) found that the effect of elevated ambient temperature on men's urolithiasis was higher than that of women. However, the incidence of stones in men was higher than that in women (7), which was mainly related to the different physiological characteristics and eating habits of men and women (29–31). However, current research still lacks an understanding of the extent of the impact of temperature. Moreover, we performed a subgroup analysis according to age and found that people aged 21–40 were more sensitive to warm and cold effects than other age groups, which was consistent with the research results of Kale et al. (32) and Wang et al. (33). It was possible that people in this age group belonged to the productive age group with more outdoor exposure, and they were more likely to engage in some heavy physical work in their daily lives, which made them more prone to dehydration (32).

Some studies analyzed the seasonal changes of patients with different urinary stone types and found that there was no obvious seasonal change trend for calcium oxalate type (34, 35), while uric acid stones frequently occurred in the third and fourth quarters of each year in Germany, with both hot and cold season (34, 35). Uric acid stones might have been more susceptible to the influence of temperature changes. However, the present study found that high and low temperatures had obvious effects on the onset of urolithiasis in Ganzhou City, where urinary stone patients were mainly calcium oxalate. Therefore, more studies in different regions would be needed to examine the influence of ambient temperature and stone type on the risk of urolithiasis.

There were several advantages to this study. First, this study collected almost all urolithiasis hospitalization data from secondary and tertiary hospitals in Ganzhou City, with large sample size and certain representativeness. Second, the DLNM model could better reflect the exposure-response relationship between the daily mean temperature and the hospitalization for urolithiasis. The confounding factors, including relative humidity and rainfall, were controlled in the model, and the research results were more reliable. Finally, the age- and gender-based stratification analysis identified those who are particularly susceptible to temperature fluctuations, which should prove helpful in the fight against diseases.

This study had some limitations. Due to the defects of ecological research, the causal relationship between ambient temperature and urolithiasis cannot be proved at present. Second, the conclusions should be carefully applied to other regions because of the diversity of the population and regions in China. Third, this study did not consider individual information, such as individual living habits and genetic factors, which might influence the estimation of the association between temperature and urolithiasis. Whatever the case, this study can be used as a reference for regions with the same level of economic development and climate.

In conclusion, both high and low temperatures could increase the hospitalization risk of urolithiasis in Ganzhou City; however, the duration of the warm effects was longer than that of the cold effects. Men and people aged 21–40 were more vulnerable to urolithiasis hospitalization related to extreme temperature. The research results will aid in the improvement of public health services under extreme temperature conditions and the implementation of more targeted interventions for vulnerable people.

## Data availability statement

The data analyzed in this study is subject to the following licenses/restrictions: The datasets generated during and/or analyzed during the current study are available from the corresponding author on reasonable request. Requests to access these datasets should be directed to YH, haoyanbing303@126.com.

## Author contributions

ZL performed the statistical analysis and wrote the first draft of the manuscript. YL participated in data cleaning and manuscript checking. XW and GL assisted with data collection and checked the results. YH contributed to the conception and design of the study and reviewed and revised the first draft. All authors read and approved the final manuscript.

## Supplementary material

Supplementary Figure 1 The spatial distribution of hospitals with admission records in Ganzhou city.

Supplementary Figure 2 The relationship between temperature and total urolithiasis hospitalizations by changing *df* for time trend.

Supplementary Figure 3 The RR and 95% CI of extreme warm (P99: 30.4°C, P95: 29.7°C) and cold (P1: 2.9°C, P5: 6.6°C) effect on the number of hospitalizations for urolithiasis at single lag day.

Supplementary Figure 4 The RR and 95% CI of extreme warm (P99: 30.4°C, P95: 29.7°C) and cold (P1: 2.9°C, P5: 6.6°C) effect on the number of hospitalizations for urolithiasis at cumulative lag days.
