# Supplementary figures and images for: Extreme temperature exposure and urolithiasis: A time series analysis in Ganzhou, China
Source: Front Public Health. 2022 Dec 14;10:1075428. doi: 10.3389/fpubh.2022.1075428 (PMC9795061; doi:10.3389/fpubh.2022.1075428)

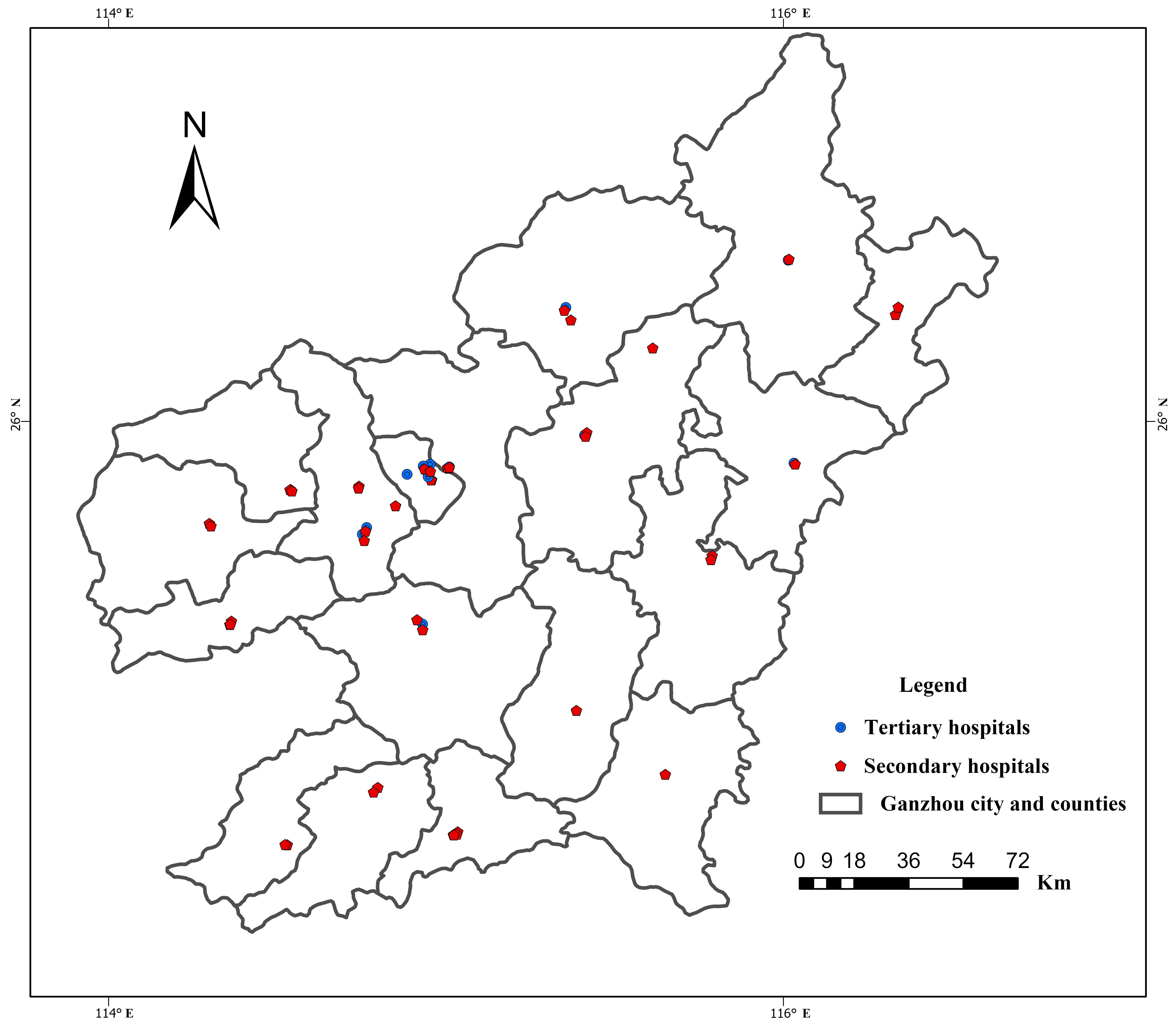

Supplement: Supplementary Figure 1 — The spatial distribution of hospitals with admission records in Ganzhou city. [file Image_1.JPEG]

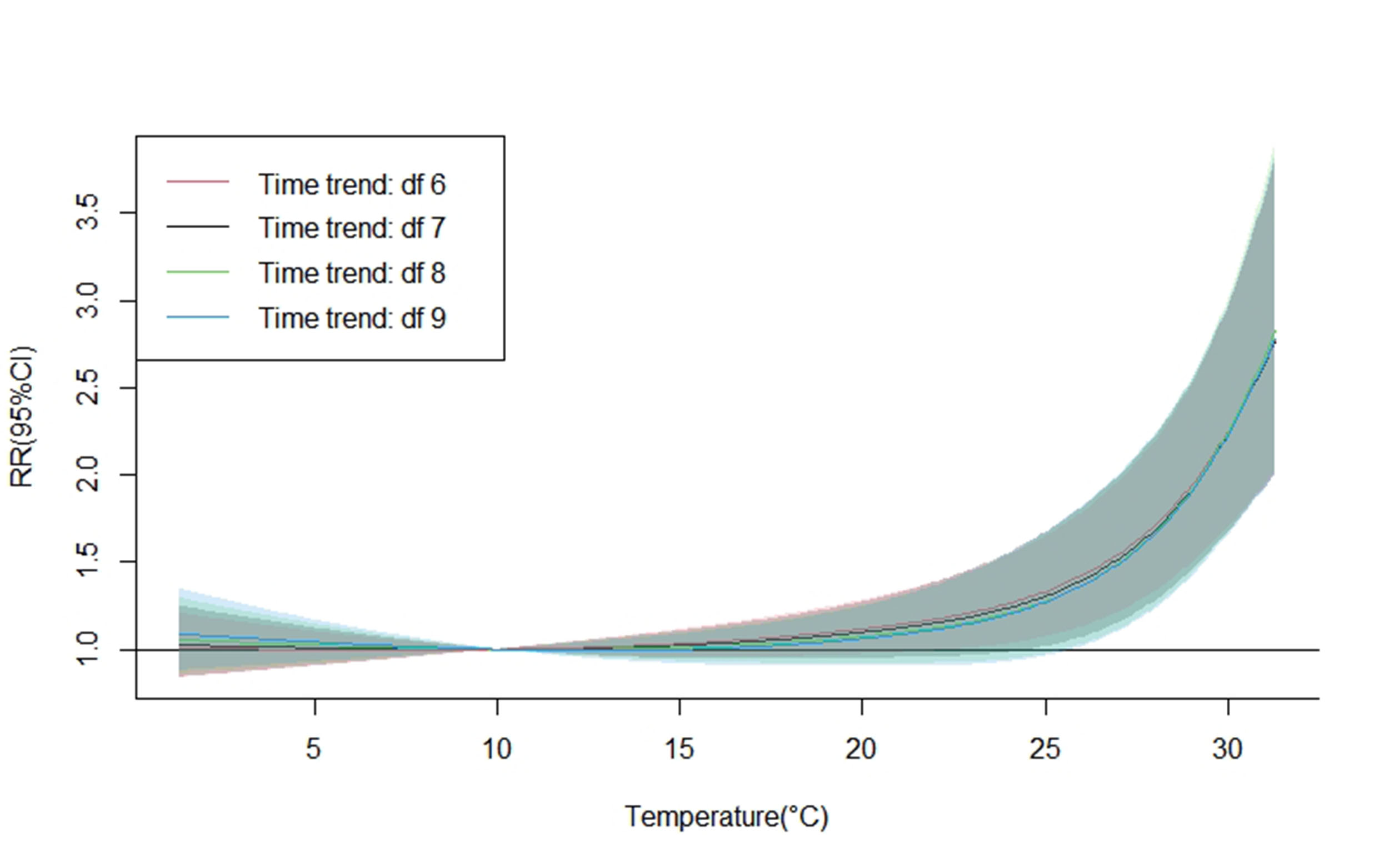

Supplement: Supplementary Figure 2 — The relationship between temperature and total urolithiasis hospitalizations by changing df for time trend. [file Image_2.jpeg]

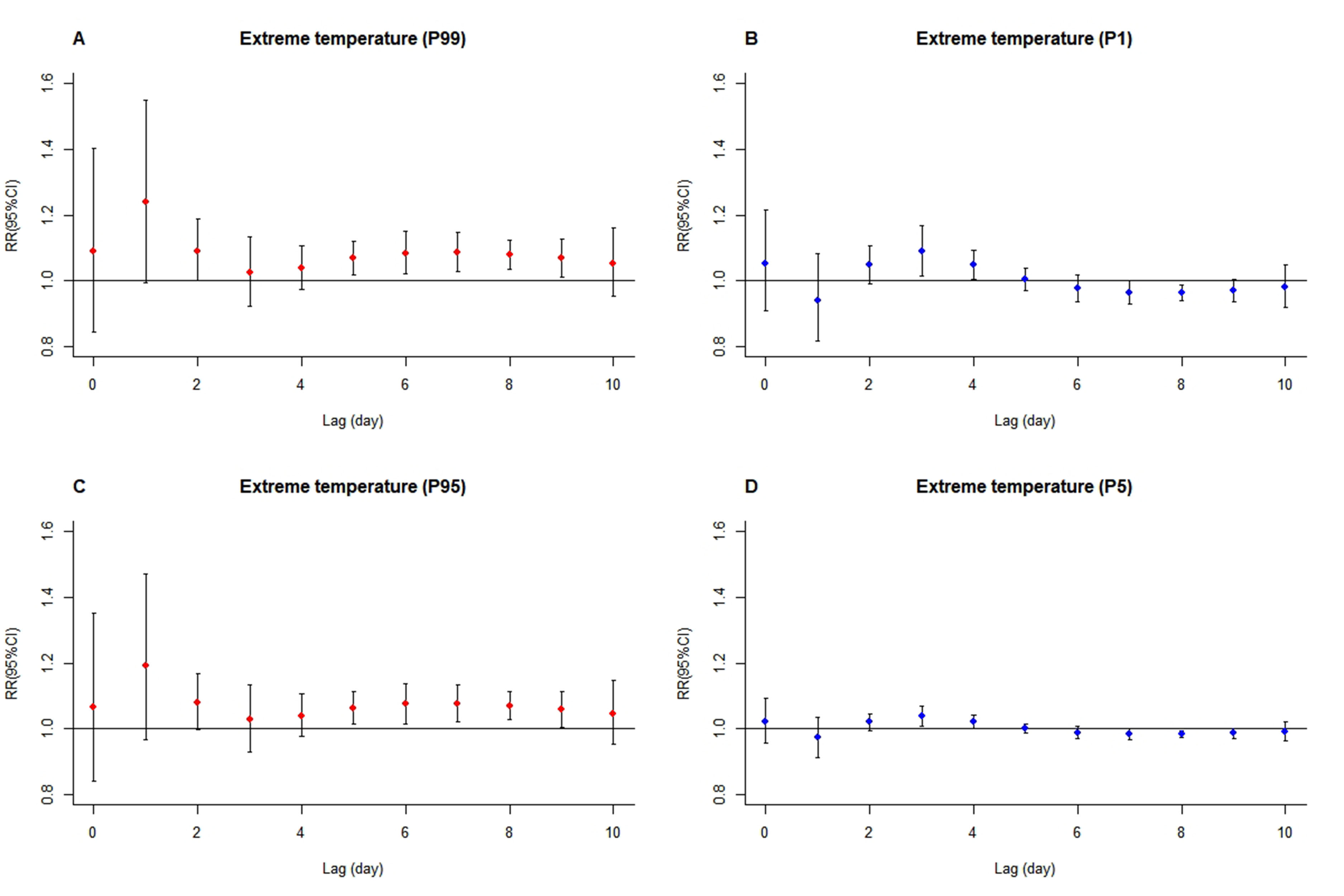

Supplement: Supplementary Figure 3 — The RR and 95% CI of extreme warm (P99: 30.4°C, P95: 29.7°C) and cold (P1: 2.9°C, P5: 6.6°C) effect on the number of hospitalizations for urolithiasis at single lag day. [file Image_3.JPEG]

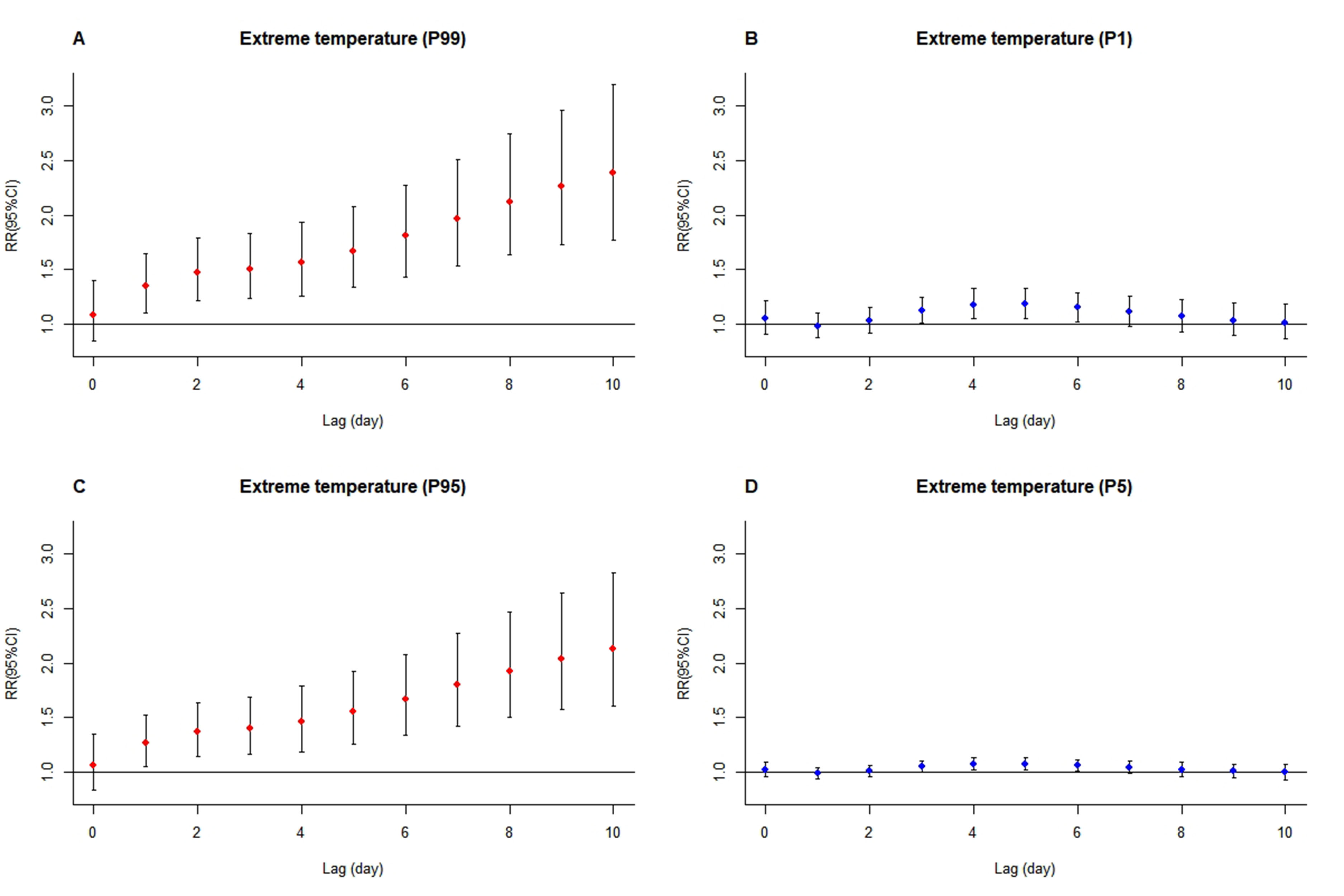

Supplement: Supplementary Figure 4 — The RR and 95% CI of extreme warm (P99: 30.4°C, P95: 29.7°C) and cold (P1: 2.9°C, P5: 6.6°C) effect on the number of hospitalizations for urolithiasis at cumulative lag days. [file Image_4.jpeg]
